# How to Efficiently Determine the Range Precision of 3D Terrestrial Laser Scanners

**DOI:** 10.3390/s19061466

**Published:** 2019-03-26

**Authors:** Berit Schmitz, Christoph Holst, Tomislav Medic, Derek D. Lichti, Heiner Kuhlmann

**Affiliations:** 1 Institute of Geodesy and Geoinformation, University of Bonn, 53115 Bonn, Germany; c.holst@igg.uni-bonn.de (C.H.); t.medic@igg.uni-bonn.de (T.M.); heiner.kuhlmann@uni-bonn.de (H.K.); 2 Department of Geomatics Engineering, University of Calgary, Calgary, AB T2N 1N4, Canada; ddlichti@ucalgary.ca

**Keywords:** terrestrial laser scanning, range precision, intensity, stochastic model

## Abstract

As laser scanning technology has improved a lot in recent years, terrestrial laser scanners (TLS) have become popular devices for surveying tasks with high accuracy demands, such as deformation analyses. For this reason, finding a stochastic model for TLS measurements is very important in order to get statistically reliable results. The measurement accuracy of laser scanners—especially of their rangefinders—is strongly dependent on the scanning conditions, such as the scan configuration, the object surface geometry and the object reflectivity. This study demonstrates a way to determine the intensity-dependent range precision of 3D points for terrestrial laser scanners that measure in 3D mode by using range residuals in laser beam direction of a best plane fit. This method does not require special targets or surfaces aligned perpendicular to the scanner, which allows a much quicker and easier determination of the stochastic properties of the rangefinder. Furthermore, the different intensity types—raw and scaled—intensities are investigated since some manufacturers only provide scaled intensities. It is demonstrated that the intensity function can be derived from raw intensity values as written in literature, and likewise—in a restricted measurement volume—from scaled intensity values if the raw intensities are not available.

## 1. Motivation

In recent years, terrestrial laser scanning has become very popular in engineering geodesy because it allows the area-based sampling of objects and the acquisition of their geometries. As the technology improves, it has become possible to use terrestrial laser scanners (TLS) for applications that require millimeter precision. Several studies use this technology in order to perform deformation analyses of, for example, water dams [1,2,3], tunnels [4] or radio telescopes [5]. However, several challenges still must be solved to achieve the goal of an area-based deformation analysis with the use of a congruency test [6,7]. So far, this has been impossible due to errors from an insufficient stochastic model that does not reflect the reality properly [7]. Therefore, a proper stochastic model in surveying tasks is necessary in order to identify outliers or to do statistical testing [8].

**Figure 1:**
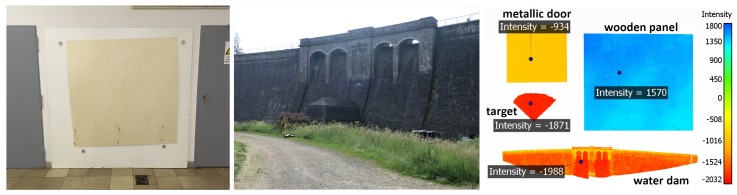
Two examples from practice [3]. **Left**: Wooden Panel; **Middle**: water dam; **Right**: Point clouds with intensity values.

Figure 1 shows two objects that were subjects of area-based deformation analyses with standard software products in [3] (Figure 1). These objects are a wooden panel and a water dam. In the point cloud of the wooden panel, there is also a gray painted metallic door and a black and white paper target. For the investigations, samples of the door and the black part of the target are considered as well. The colors in Figure 1 (right) indicate the intensity values of the points. It is obvious that varying reflectivities cause different intensity values. This is important, as it is demonstrated in [9] that the variations of the range precision, evoked by different scan configurations and object reflectivities, can be modeled as a function, which depends solely on the intensity value. Furthermore, the relation between range precision and intensity can be modeled with an exponential function. This function can be incorporated into the stochastic model in order to make it more realistic. Hence, the objects of Figure 1 have different range precisions. The goal of this study is to find an efficient and economic procedure to calculate a range precision for the points of the presented examples and to investigate whether this could also be done with scaled intensity values since previous studies only dealt with raw intensities. Generally, the range noise needs to be quantified for each scanner and furthermore, for different settings, such as different scan rates, quality levels and laser powers.

Previous studies presented approaches to determine the range precision of TLS. However, these approaches are not applicable to every scanner. In [9], the range precision is derived from multiple measurements of one point with a TLS that operates in 1D mode, which is not applicable for every scanner and without permission of the manufacturer. Approaches to tackle this problem, by using the residuals of a best fit plane, are given in [10,11].

Additionally, raw intensity values were used. They contain physical information about the strength of the reflected signal. Not many software products provide the actual raw intensity values. One of them is Z+F LaserControl. Some manufacturers scale their intensity values, for example, to improve the visual appearance or to simplify segmentation tasks [12,13,14,15]. Leica Cyclone, for instance, scales the intensity values between −2047 and 2048 [15] in a .pts-file or between 0 and 1 for example in a .ptx-file. Not all manufacturers provide the conversion between raw and scaled intensity. Hence, it is not exactly known in which way the scaling happens. One possible option would be that the maximum received signal gets the value 2048 and the lowest signal gets the value −2047. This would be a relative scaling. Another option could be that a certain raw value always gets the same scaled value, which would be an absolute scaling. This will be part of the investigations in Section 6.

Hence, these contributions are not sufficient for the previously presented applications as they were sampled with a Leica ScanStation P20 that does not operate in 1D mode, and Leica Cyclone does not supply the raw intensity values. For this reason, this study focuses on the following issues:

- An efficient approach will be presented which allows qualified users to determine the range precision of their terrestrial laser scanners from 3D points. The setup should be cheap and easy to replicate. So, it can be easily performed for several scanner settings (Section 5).
- As not every manufacturer provides raw intensity values, the second goal of this study is to find out whether raw as well as scaled intensity values, such as those from Leica Cyclone, can be utilized for determining the range precision of the given examples (Section 6).

Achieving the previously mentioned aims, a function for the Leica ScanStation P20 can be derived in order to calculate the range precision of the examples in Figure 1. Besides the Leica ScanStation P20, the Leica HDS6100 is also examined in more detail. This is necessary because raw intensity values can be obtained for the Leica HDS6100 but not for the Leica ScanStation P20. The comparison of raw and scaled intensity values is indispensable as it must be clarified whether both kinds of intensity values can be utilized for the range precision modeling.

Therefore, Section 2 recapitulates previous studies on this topic. Section 3 explains the proposed methods and Section 4 describes the experiments that are carried out in this study. Section 5 and Section 6 deal with the previously mentioned issues. In the end, the results are discussed in Section 7, and Section 8 shortly summarizes the achievements of this study.

## 2. Previous Work

As reported in several studies, the accuracy of TLS measurements depends on multiple factors. Soudarissanane et al. (2011) [16] pointed out the main four influences on a TLS measurement as instrument mechanism, atmospheric conditions, object surface properties and scan geometry. They affect the rangefinder, which will be examined in this study. Especially for shorter distances, the rangefinder plays the most important role regarding the 3D point accuracy (e.g., [17]).

Different aspects influence the rangefinder accuracy, which have a systematic background. Several authors discovered that the offset in distance measurements is related to the target reflection properties (for example [18,19,20]). In the latter study, it was concluded that targets with high reflectivity are measured farther away than targets with low reflectivity, even though they are installed at the same position. The magnitude of this offset can reach up to several millimeters. However, the object surface should not be neglected likewise as it characterizes how the signal, that has been emitted by a scanner, is reflected. Hence, the reflectivity is a property of the object surface. Zámečníková and Neuner (2017) [21] found out that a combined influence of incidence angle and surface roughness exists. However, these findings are not further investigated as this paper only focuses on measurement precision and not accuracy.

In order to model the precision of the rangefinder of a TLS, the deteriorating influences have to be well known. Several previous studies focused on the quantification of random errors by using the residuals of a best fit plane [16,22,23] or a sphere [23,24]. Soudarissanane et al. (2011) [16], for example, investigated the influence of the scanning geometry on the point cloud. They found that the signal-noise-ratio (SNR) decreases with higher incidence angles. This is also related to the reflectivity of the surfaces since they showed that the intensity decreases with higher incidence angles. Several other studies also investigated the relationship between noise of TLS, the intensity and the scan configurations, e.g., [9,10,11,13,19,25]. It is always obvious that a relation between all those aspects exists. However, so far, no approach exists to model all mentioned influencing factors individually.

Wujanz et al. (2017) [9] summarized these findings by establishing the functional relation between the precision of the rangefinder of TLS and its raw intensity values, which covers the influence of different ranges as well as different incidence angles and surface properties. These investigations are based on multiple samples of the same point measured in 1D mode (i.e., only range measurement), which cannot be performed with a common panoramic-type TLS.

For this reason, Lambertus et al. (2018) [10] did an empirical investigation to prove the suitability of the intensity-based range precision in the 3D Euclidean space. They investigated the root-mean-square error of a plane fit depending on the intensity value. Wujanz et al. (2018) [11] likewise obtained the range precision by using residuals of a plane estimation, which are orientated in surface normal direction because all measurement elements are weighted equally. In both studies, the methods are only applicable to measurements with zero-degree incidence angle because otherwise the influence of the angular encoders is too high. Furthermore, Wujanz et al. (2018) [11] tested the transferability of the model to other rangefinder types as, so far, only phase-based laser scanners have been the focus of the investigations. Therefore, they used a Riegl VZ-400i impulse scanner and a Leica ScanStation P40 TLS, which uses a hybrid rangefinder technology. This study showed that the other scanners also follow the established functional relation between intensity and range precision if the raw intensity values are known.

Heinz et al. (2018) [26] introduced an approach to model the range precision of the 2D laser scanner Z+F Profiler 9012A. Therefore, the static scans of surfaces with different backscatter are used, and the range precision is estimated from numerous overlapping scan profiles. This method does not rely on the assumption of a geometric primitive but it requires a rather planar surface.

The four latter mentioned studies only dealt with raw intensity values, which are not available for all scanners. Theoretically, the received laser power should follow the rules of the radar range equation introduced by [27]

(1) $$\begin{array}{c} P_{r}=\frac{P_{t}D_{r}^{2}\rho }{4r^{2}}\eta_{sys}\eta_{atm}\cos\left(\alpha \right), \end{array}$$

where the received laser power $P_{r}$ is a function of the transmitted laser power $P_{t}$, the receiver aperture diameter $D_{r}$, the range *r*, a system factor $\eta_{sys}$ and atmospheric transmission factor $\eta_{atm}$, the target reflectance ρ and the incidence angle α. This function varies using different reflective surfaces, ranges, incidence angles, atmospheric conditions and different scanners. The explanation as to why the range precision can be modeled with the received laser power is given in [11]. They explain why the functional relation is capable of reflecting the random characteristics of a reflectorless rangefinder. However, this function does not always reflect the actual intensity values as some manufacturers amplify the received signal with the distance [12,14,28,29,30]. Even though the latter studies did investigations on the radiometric calibration of the intensity, the scaling processes of the instruments used in this study are not revealed. Hence, information about the influence of the distance on the scaled intensity is not given.

## 3. Methodology to Model the Range Precision with 3D Points

The computation of a standard deviation of the range measurements is not straightforward with a scanner that measures in 3D mode because for most terrestrial laser scanners, it is impossible to measure the same point multiple times. Some scanners can measure in 1D range mode, but this mode is usually not accessible due to health and safety regulations. Thus, the procedure of estimating the observation noise for this kind of scanner is outlined in Section 3.1. Section 3.2 recapitulates the estimation of the range precision depending on the intensity, which was introduced by [9].

### 3.1. Determination of the Range Precision of 3D TLS

In order to obtain a function, which models the relation between intensity and range noise, it is necessary to calculate the range precision from different test samples. For this purpose, the residuals of a plane adjustment are used comparable to [11]. The computations of the plane adjustment are carried out according to the description of [31]. Since the elements of the TLS measurement $l={\left[r,\varphi ,\theta \right]}^{T}$ are range *r*, horizontal angle φ and vertical angle θ, a plane in the three dimensional space is described by the following equation:

(2) $$\begin{array}{c} n_{x}\cdot r\cdot \sin\theta \cdot \cos\varphi +n_{y}\cdot r\cdot \sin\theta \cdot \sin\varphi +n_{z}\cdot r\cdot \cos\theta -d=0. \end{array}$$

In this equation, $n_{x},n_{y}$ and $n_{z}$ are the components of the unit normal vector n of the plane and *d* is the orthogonal distance between origin and plane.

The plane is estimated using a Gauß-Helmert model [8]. The whole procedure is described in [31]. It needs to be emphasized that, different to [10,11], the single measurement elements are not weighted equally. The covariance matrix $\Sigma_{ll_{P}}$ of the observations is built as follows:

(3) $$\begin{array}{c} \Sigma_{ll_{P}}=\left[\begin{array}{cccc} \sigma_{r_{1}}^{2} & & & \\ & \sigma_{\theta_{1}}^{2} & & \\ & & \sigma_{\varphi_{1}}^{2} & \\ & & & \ddots \end{array}\right]. \end{array}$$

Initial values are taken from manufacturers’ specifications, and they are modified in an iterative adjustment with variance component estimation [8]. The measurement components are assumed to be uncorrelated as usual since the correlation is not known [32].

Residuals of the ranges ($v_{r}$), the horizontal and vertical angles ($v_{\varphi }$, $v_{\theta }$) are obtained from the adjustment. Consequently, the range precision can be directly estimated from the range residuals

(4) $$\begin{array}{c} \sigma_{r}=\sqrt{\frac{1}{n}\sum_{i=1}^{n}v_{r_{i}}^{2}}. \end{array}$$

It follows that this method can be applied to scans which do not have a zero-degree incidence angle as the range residuals are aligned in laser beam direction and not perpendicular to the plane when the single observation groups are not weighted equally.

### 3.2. Modeling the Intensity-Based Range Precision

Having estimated the range precision of several test samples, the standard deviation of the range measurement can be modeled. As it is demonstrated in [9,11], the following model covers all influences that cause random errors: Equation (5) is the base for fitting a function which estimates the range precision dependent on the intensity value [11]:

(5) $$\sigma_{r}=a\cdot Int^{b}+c.$$

Here, *Int* describes the intensity values; *a*, *b* and *c* are the unknown parameters, which define the function; and $\sigma_{r}$ is the standard deviation of the range. In literature, Equation (5) is used with and without parameter *c* [11]. Therefore, its significance will be tested.

After estimating the noise level, the parameters of Equation (5) are estimated using a Gauß-Markov model [8] in order to obtain a function for the range precision. Herein, intensity values are treated as constants and the range precision as observations. To test the compatibility between the observations and the model, a global test is carried out after the adjustment. On that account, the estimator for the variance factor $s_{0}^{2}$ is tested against the theoretical variance factor $\sigma_{0}^{2}$ [8]. If the global test is rejected, the stochastic model is modified by substituting $\sigma_{0}^{2}$ with $s_{0}^{2}$, and the adjustment is carried out again until it passes. If it passes, the single parameters of Equation (5) are tested for significance. Since the parameters are correlated, they cannot be considered as independent parameters; hence, they need to be decorrelated before the statistical testing. Therefore, as described in [33], a Cholesky-decomposition is used to obtain uncorrelated parameter values.

In order to examine how the functional relation of Equation (5) fits to the data, the coefficient of determination *B* is computed [8] as

(6) $$\begin{array}{c} B=\frac{l^{T}l-v^{T}v}{l^{T}l}. \end{array}$$

The value for *B* is between 0 and 1, where 1 means that the given functional relation completely explains the variations of the observations **l**. The residuals of the observations are described by the variable **v**.

## 4. Experiments

In order to determine the range precision of the Leica HDS6100 and the Leica ScanStation P20 TLS, it is necessary to collect several data. Comparing the range measurement technology, the difference between both scanners is that the HDS6100 uses a phase-based method [34] to measure distances, whereas the P20 uses a time-of-flight enhanced by wave form digitizer (WFD) technology [35].

Section 4.1 describes the data collection with the so-called Spectralon targets, which are professional diffuse reflecting targets. Afterwards in Section 4.2, a measurement setup is presented which is built of paper targets, which is used for an efficient determination of the range precision. Section 4.3 describes the data collection to investigate scaled intensities. In all experiments, the planarity of the chosen samples is superior to the expected values of the range precision.

### 4.1. Data Collection with Spectralon Targets

**Figure 2:**
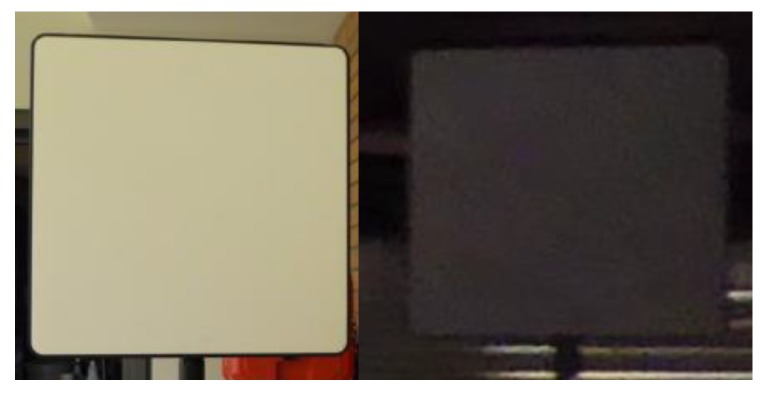
Spectralon targets (26 cm × 26 cm) with high reflectivity (**left**) and low reflectivity (**right**).

In the first experiment, professional diffuse reflecting targets, known as Spectralon targets, were used. Two different Spectralon targets (26 cm × 26 cm) with very high and very low reflectivity (Figure 2) were scanned with the Leica HDS6100 TLS with varying distances and incidence angles. The experiment took place in a basement in order to ensure that no ambient light influenced the measurements. Furthermore, the indoor environment was temperature controlled.

The targets were placed at 11 different distances (2 m, 3 m, 4 m, 5 m, 6 m, 8 m, 10 m, 12 m, 16 m, 20 m and 30 m). Because of the limited space in the basement and the size of the targets, no longer distances have been investigated. The heights of the target and of the scanner were the same to ensure a vertical angle of 90 degrees. Additionally, to establish a horizontal incidence angle of zero degree, the targets had to be aligned perpendicular to the laser beam direction. This was realized with a Leica TS30 total station by measuring the distance to the two edges of the target with $90^{\circ }$ vertical angle. Each target was rotated until the difference of both range measurements was less than 1.5 mm.

In another setup, the distance between device and target remained the same, but the incidence angle changed from $0^{\circ }$ to $70^{\circ }$ with $10^{\circ }$ increments. Therefore, the position of the target remained the same, but the instrument was moved on a circle with a radius of 6 m and angular increments of $10^{\circ }$. In order to avoid any effects in the rangefinder due to an error in the vertical or horizontal angle encoder, the target and the device were again installed at the same height, and the horizontal angle remained approximately the same. Subsequently, the targets were sampled with the previously mentioned terrestrial laser scanner. All scans were collected with a sampling rate of 508 kHz, which leads to a point spacing of 3.1 mm × 3.1 mm at 10 m range [34].

### 4.2. Data Collection with Paperboards

In the next experiment, a measurement setup is presented which should allow qualified users of laser scanners to analyze their range precision without the need for special targets that only reflect diffuse. Three demands are imposed on the setup:

- Firstly, it needs to be built with little effort and it should be cheap.
- Secondly, the precision still has to be determinable.
- Lastly, a wide range of intensity values needs to be obtainable.

As not every user has professional reflecting targets, the setup is made of paper targets, which partially reflect specular. Consequently, the backscattered signal is still strong even when dark surfaces are used. For this reason, the setup is also measured with higher incidence angles as the intensity decreases with higher incidence angles [30]. This is necessary in order to reach low enough intensity values for the modeling of the relation between range precision and intensity. The planarity of the targets is superior to the TLS precision.

**Figure 3:**
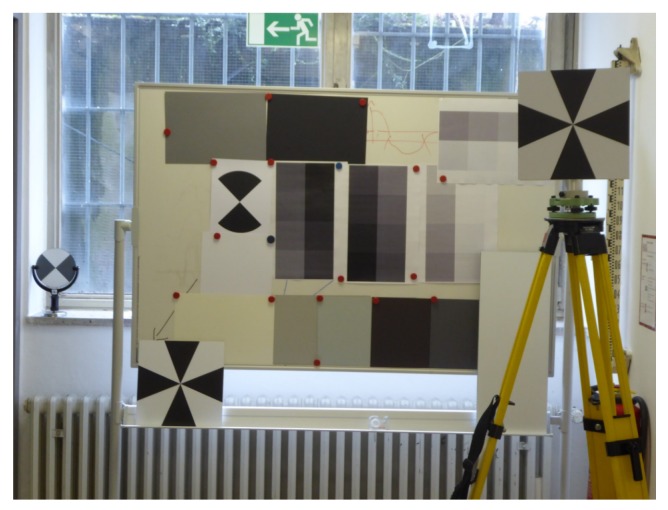
Data collection with targets of different gray scales.

Figure 3 presents the setup which was made of black and white targets, cardboards with different shades of gray and different gray scales printed on a sheet of paper. All these targets are fixed to a magnetic wall. In an earlier setup, different colors were used as targets, but the result of the examination was that colors do not have the same influence on the reflectivity as targets with different gray scales. Thus, there was not enough variety in the intensity values. Consequently, a new setup was built with gray scales. The targets were scanned several times with incidence angles of $10^{\circ }$, $25^{\circ }$, $40^{\circ }$ and $50^{\circ }$ and distances of 8 m and 22 m. This time, the data collection was carried out again with a Leica HDS6100 TLS, but not with the same instrument as before and with a Leica ScanStation P20.

### 4.3. Data Collection to Analyze Scaled Intensities

**Figure 4:**
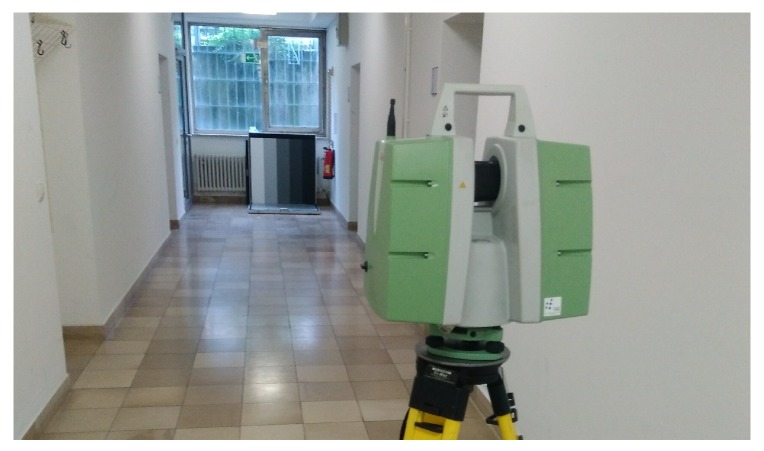
Experimental setup to examine reproducibility of intensity values.

In order to verify whether the scaled intensity values are constant and not scaled relatively, additional measurements were taken with the Leica ScanStation P20. As shown in Figure 4, a diffuse reflecting Spectralon target (1 m × 1 m) was used. The target is divided into five different gray scales, which are used as planar areas with the same reflectivity from which a range precision can be calculated for each part. All scans were taken with a resolution of 0.8 mm @ 10 m at a distance of 15 m. To investigate the influence of different distances on the range precision estimated from scaled intensities, the target was scanned again from 35 m, 50 m and 75 m with the Leica ScanStation P20 with a resolution of 0.8 mm @ 10 m and the quality level of 1. These distances are chosen as they are mainly used in engineering geodesy.

In order to investigate whether the intensity changes during multiple measurements or after restarting the instrument, five scans of the same setup at a distance of 15 m were taken in a row. Furthermore, the instrument was turned off and turned on again, and the scan was carried out again. Afterwards, the battery was changed, and the setup was scanned again. Lastly, only the black part and then only the white part of the target were sampled in order to check whether the scaling depends on the spreading of the maximal and minimal raw intensity value within each scan. The measurements were collected right after each other and the external conditions remained constant during the experiment.

## 5. Efficient Modeling of the Range Precision with 3D Points

In this section, the range precision of the Leica HDS6100 TLS is modeled according to the methods explained in Section 3. In Section 5.1, the data that are collected of the Spectralon targets are analyzed and a function which models the relation between intensity and range precision is estimated. Afterwards in Section 5.2, the data that are collected with the paperboards are investigated.

The estimation of the range precision requires some preprocessing, which is done before the analysis: In order to estimate the range precision of the collected samples, points that belong to the same target are cut and a mean intensity is calculated. Additionally, as it is assumed that these points lie on a plane, a plane is estimated as described in Section 3.1. Afterwards, the range precision is derived from the range residuals of the plane adjustment (Equation (4)). Furthermore, the incidence angle for each point is computed as described in [16] and the mean incidence angle of the sample is calculated afterwards.

### 5.1. Determination of Intensity-Based Range Precision with Sepctralon Targets

**Figure 5:**
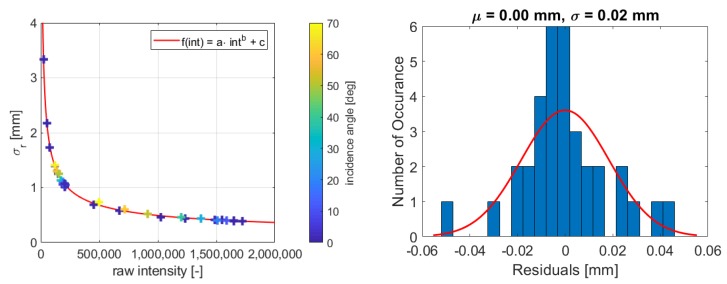
**Left**: Estimated function for HDS6100 and scan rate of 508 kHz; **Right**: Corresponding histogram of the residuals of the function.

This section investigates the samples of the Spectralon targets (Section 4.1). The range precision is calculated for each sample and Figure 5 shows the estimated function from Equation (5) on the left with the corresponding histogram of the residuals on the right. It is visible that all samples fit the function very well, which is confirmed by the coefficient of determination of B = 0.99 (Equation (6)). The residuals are distributed around zero with a maximum deviation of 0.02 mm. Due to the limited number of 34 observations, the histogram does not strictly follow a Gaussian distribution, but no significant systematic deviations are visible.

In Figure 5 (left), the different colors indicate the different incidence angles. It is obvious that samples with high incidence angles also fit the function as they are not deviating more than the samples that are taken with zero-degree incidence angle. In [11], it is demonstrated that this method works with samples that are aligned perpendicular to the scanner. This study shows that samples with higher incidence angles do not deviate from the other samples as the magnitude of the residuals is not higher than the magnitude of the residuals with zero-degree incidence angle. Both types of residuals are distributed around zero with the same order of magnitude. Hence, it is concluded that the incidence angle does not have a substantial influence on the function. This implies that the method presented in this paper is capable of dealing with high incidence angles if the measurement components are not weighted equally in the plane adjustment as explained in Section 3.1. Consequently, the model is capable of modeling both the influence of the range and the influence of the incidence angle.

### 5.2. Determination of Intensity-Based Range Precision from Paperboards

This section considers scans of the paperboard setup of Section 4.2. Again, the range precision and the function to model range precision and intensity are estimated. As shown in Section 5.1, it is possible to use scans with higher incidence angles than zero. This is very beneficial as the paper targets are partially reflecting specular and hence, a higher intensity is measured. Rotating the targets leads to lower intensity values, which is necessary for a proper fitting of the function.

**Figure 6:**
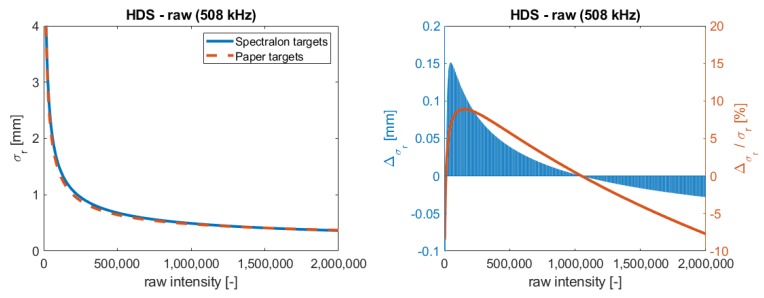
Comparison of functions estimated with Spectralon targets and with the paperboard setup.

In Figure 6, the resulting function is compared to the function that was estimated in Section 5.1. The left plot shows the functions and the right plot the corresponding difference between them including its percentage share. Comparing the two functions, they look almost similar. A slight deviation in the curviest part of the function is visible. However, considering the differences on the right in Figure 6, it is visible that the functions vary and that the deviation is systematic. Especially, for small intensities, the values increase up to 0.15 mm. This results from the empirical data set with different scanners and the uncertainties that are yielded from computing the mean intensity values for one sample. Hence, the functions have uncertainties, which cause deviations in the function. Nevertheless, the largest deviation is smaller than 10% of the range precision. This implies that, with a tolerance of 10%, the function can also be estimated from a setup, which is not associated with high costs and which is easy to install. Furthermore, the function is reproducible for this scanner type and for these different setups.

## 6. Investigations of Raw and Scaled Intensities

The previous investigations used raw intensity values and scans of the Leica HDS6100. Having a Leica laser scanner, Cyclone is primarily used for point cloud post-processing, which scales the intensity. With the idea to estimate the stochastic model in the most practical manner, the Cyclone intensities are now investigated further in order to estimate a range precision for the applications that were mentioned in the motivation (Section 1).

To get an impression of the differences between raw and scaled intensity values, Section 6.1 investigates the behavior of the intensity with different incidence angles and distances. In Section 6.2, the range precision of the Leica HDS6100 and of the Leica ScanStation are estimated depending on the scaled intensities of Cyclone. Afterwards, Section 6.3 examines whether the scaled intensity values are constant or whether they are scaled relatively to the rest of the point cloud as it is mentioned in Section 1. This is indispensable because otherwise, the shape of the function would change in each scan. Furthermore, the influence of the distance on the function is examined in Section 6.4.

### 6.1. Relation between Intensity, Distance and Incidence Angle

**Figure 7:**
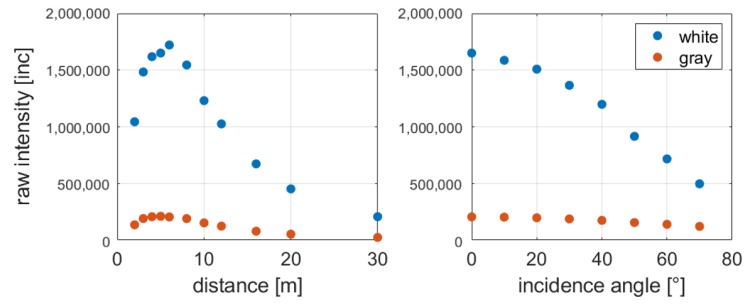
**Left**: Relation between raw intensity and range; **Right**: Relation between raw intensity and incidence angle for the Leica HDS6100.

As seen in Equation (1), the strength of the reflected signal strongly depends on the range and on the incidence angle. These two parameters are chosen for investigation because the others almost remain constant while measuring with the same target and instrument. Figure 7 shows the relation between distance and raw intensity on the left as well as incidence angle at a distance of 6 m and raw intensity on the right. These results are obtained from the measurements of Section 4.1.

It is obvious that the strength of the received signal increases with longer distances up to a distance of six meters. Afterwards, the intensity decreases. This effect is caused by a shadowing effect on short distances. The aperture for the emitted laser beam is located in the center of the received laser beam and in front of the avalanche photodiode. Hence, the aperture causes a shadow in the cross-section of the laser beam, which grows larger with shorter distances. Consequently, less signal reaches the avalanche photodiode. A detailed explanation is given in [26].

Furthermore, it is visible that the intensity decreases with increasing incidence angle as it is predicted in the radar range equation (Equation (1)). The reflected signal from the white surfaces of the Spectralon target decreases faster than that of the gray surface. Additionally, the reflectivity of the gray target abates slower because the intensity is much lower in the beginning and cannot decrease that much anymore.

**Figure 8:**
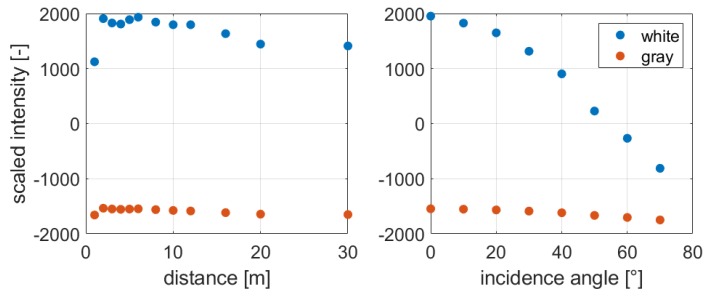
**Left**: Relation between scaled intensity and range; **Right**: Relation between scaled intensity and incidence angle for the Leica HDS6100.

Figure 8 shows the relation between scaled intensity and range (left), and scaled intensity and incidence angle (right) for each Spectralon target, scanned with the HDS6100.

Considering the relation between intensity and range, usually, the intensity decreases with increasing distances (Equation (1), Figure 7). However, as shown in Figure 8, this is not the case for scaled intensities. Especially on the first 12 m, a slight variation is visible, but subsequently, the intensity almost stays constant. This implies that the intensity is amplified distance-dependent by the manufacturer during the scaling process.

As the intensities of the gray target do not decrease as much as the ones of the white target, the higher intensity values are more influenced by the amplification. Considering the function in Figure 5, higher intensity values move on the almost constant part of the function if they are manipulated by the manufacturer. On the contrary, the impact on the position of low intensities in the function would be affected much more. However, fortunately, low intensities are less influenced by the amplification during the scaling process. Hence, this could be beneficial for the estimation of the range precision with scaled intensity values.

Regarding the relation between intensity and incidence angle, no valuable difference in the behavior of the intensity is visible compared to Figure 7.

Hence, it is clear that scaled intensities cannot follow the function established by [9] as already pointed out in [9,11]. Since the intensities are amplified with the distance, the intensity does not cover the influences of incidence angle and distance on the range precision. Nevertheless, the next sections investigate whether there is any possibility to estimate the range precision with scaled intensities at least for limited ranges.

### 6.2. Estimated Function with Scaled Intensities

**Figure 9:**
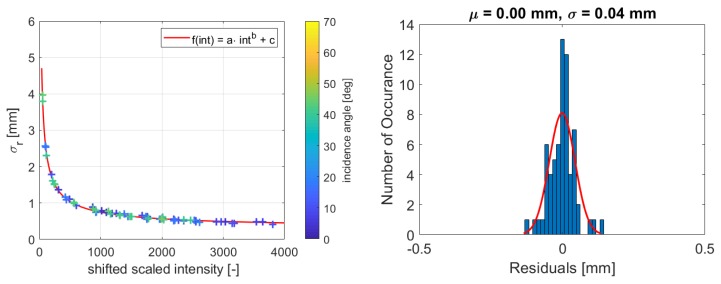
**Left**: Estimated function for Leica HDS6100 with a scan rate of 508 kHz; **Right**: Corresponding histogram of the residuals of the function.

**Figure 10:**
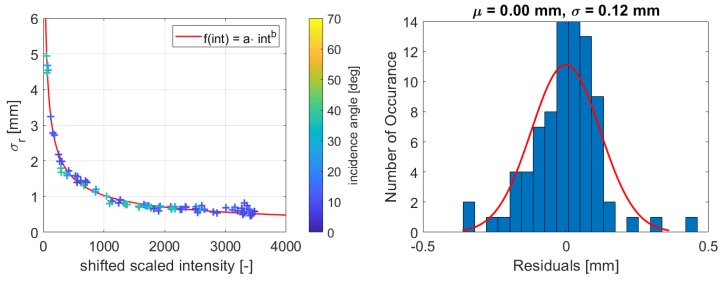
**Left**: Estimated function for Leica ScanStation P20 and resolution of 0.8 mm @ 10 m; **Right**: Corresponding histogram of the residuals of the function.

**Figure 11:**
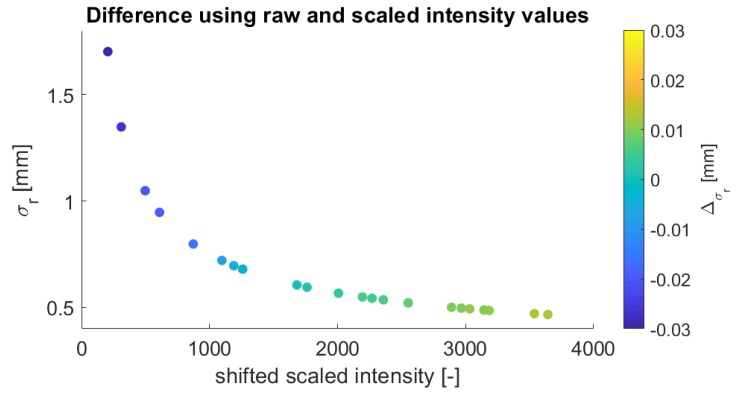
Comparison of the range precision calculated with the raw and scaled intensity values by inserting these values in the estimated function.

Scans of the new measurement setup from Section 4.2 are taken with the Leica HDS6100 with a scan rate of 508 kHz and the Leica ScanStation P20 with a resolution of 0.8 mm @ 10 m and quality level 1. The scaled intensity values from Leica Cyclone are used to model the range precision of both instruments dependent on the scaled intensities. Since the adjustment only allows positive intensity values, the intensities are shifted by adding 2050 to the original value. For this reason, the x-axes in Figures 9–11 and 13–15 are labeled with shifted scaled intensity. Samples of measurements with 8 m and 22 m distance between scanner and targets as well as incidence angles of 10${}^{\circ }$, 25${}^{\circ }$, 40${}^{\circ }$ and 50${}^{\circ }$ are considered in the estimation.

Figure 9 and Figure 10 present the estimated functions for both scanners with the corresponding histograms of the residuals. Comparing the figures, the precision of the samples measured with the HDS6100 notably fits better to the function. This can be likewise concluded from the histograms, as the standard deviation of the distribution fit is much better for the Leica HDS6100.

Nevertheless, this distribution fit also shows that no bias exists and that the residuals are distributed randomly around zero, which allows the execution of the adjustment with the given data sets. Hence, it is reasonable to fit the function for both scanners, and considering the coefficient of determination (Equation (6)), which is B=0.99 for both scanners, the function suits well to the dataset. This is especially important for the left part of the function where its variation is the highest. Hence, for both scanners, this function can be properly estimated.

To verify these functions, the estimated range precision between both intensity types will now be compared. To examine that, the precision of the same dataset collected with the Leica HDS6100 is once estimated with raw and once with scaled intensity values by inserting the values in the function with the calculated parameters to get a direct comparison. Figure 11 shows the difference between both kinds of intensity.

The maximum absolute deviation amounts to approximately 0.03 mm at the lowest intensity value. The absolute difference gets smaller with higher intensity, and it increases again at an intensity value of almost 0. This is predictable as the function has much more variation in the low intensity part. Meaning, a slight difference in the scaling of the intensity value will be visible the most in the lower intensity range. However, this implies that both functions are crossing each other at the border of negative and positive deviations and that the deviations are systematic. As they are both obtained empirically from noisy data, it can happen that there is a small deviation in the function, but the magnitude is so small that it is negligible.

The estimated precision from the function for the corresponding intensity value of the largest absolute deviation in Figure 11 is 1.78 mm. Consequently, the maximum difference between raw and scaled intensity means 1.7% of the estimated precision from the function. As this value is very small, it is assumed that this difference does not have a significant influence on the function. Following, it indicates that the functional relation can also be modeled with the scaled intensity values for this data set.

### 6.3. Reproducibility of Intensity Values

**Figure 12:**
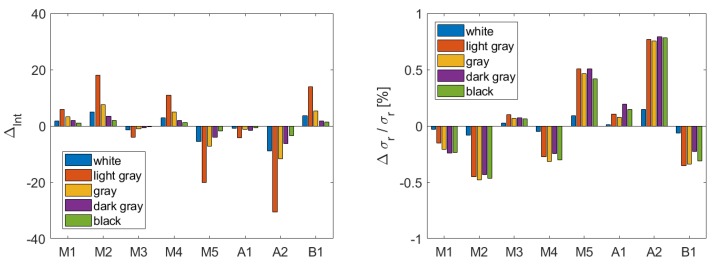
**Left**: Difference between intensity values of measurement one and those from the other measurements at a distance of 15 m; **Right**: Percentage of the corresponding differences of the range precision. M1-M5: Measurements, that were taken one by one; A1, A2: Measurements after restarting the instrument; B1: Measurement after changing the battery.

For the estimated function, which models the relation between range precision and intensity, it is essential that the measured intensities stay constant under the same conditions. To prove this, the measurements from Section 4.3 are examined. The mean intensity is calculated for each panel of the big diffuse reflecting target (Figure 4). Afterwards, the difference between the first measurement and the others is computed, and it is visualized in Figure 12 (left). Furthermore, the corresponding difference of the range precision is calculated by inserting the inherent intensity value in the estimated function from Section 6.2. The percentage of the difference from the corresponding value in the function is visualized in Figure 12 (right) as well. M1–M5 denote the measurements that are carried out one by one, A1 and A2 describe the measurements that are taken after restarting the instrument and B1 shows the measurement after changing the battery.

It is obvious that there is either a positive trend or a negative trend for the differences of the intensity values of the same measurement. This implies that the received signal slightly differs between the measurements. However, the sign and the magnitude also differ between the measurements. Following, there is no systematic trend visible during all measurements. This is also valid after restarting the instrument or changing the battery.

The biggest difference is visible for the second brightest panel (light gray). The smallest difference is obtained for the panel with low reflectivity (black). The function, which models the relation between range precision and intensity, has the largest variations for low intensities. However, the resulting differences of the range precision are less than 1% of the actual values in the function for the inherent intensity, which is very small and hence, negligible.

Furthermore, the investigations did not reveal any differences when only one part of the target was scanned. Hence, the intensity can be assumed constant while measuring on equal terms. This conclusion confirms the utility of the function. It follows that the function can be determined one time, and it can then be used for other measurements with different scan configurations.

**Figure 13:**
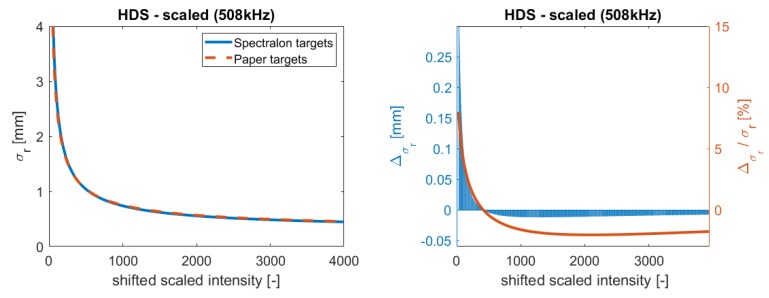
Comparison of functions estimated with Spectralon targets and with the paperboard setup using scaled intensity values.

Hence, it can be concluded that the function is reproducible at least if the same ranges are used. Figure 13 shows the two functions estimated from Spectralon targets (setup from Section 4.1 considering measurements with distances up to 20 m) and estimated from the paperboard setup (Section 4.2) for the Leica HDS6100. That means both functions are estimated from different data sets that were collected in different labs, with different targets and with different scanners from the same type. The resulting differences amount to less than 10% for very low intensities (lower than −2000). For higher intensities the deviation is less than 5%, which is lower than the difference when raw intensity values are used (Figure 6). To conclude, this section demonstrates that intensity values and the function itself can be reproduced, which justifies the use of scaled intensity values.

### 6.4. Influence of the Distance on Scaled Intensities

So far, the modeling of the range precision works out with scaled intensities with scans that are collected from distances up to 22 m. As already mentioned in Section 2, the scaled intensity values are assumed to be amplified with the distance by the manufacturer. In the previous investigations, where distances up to 22 m were used, a deteriorating effect is not visible. Nevertheless, this will now be investigated for longer distances. In order to check whether this effect influences the modeling of the range precision, longer distances from the setup of Section 4.3 are taken into account.

**Figure 14:**
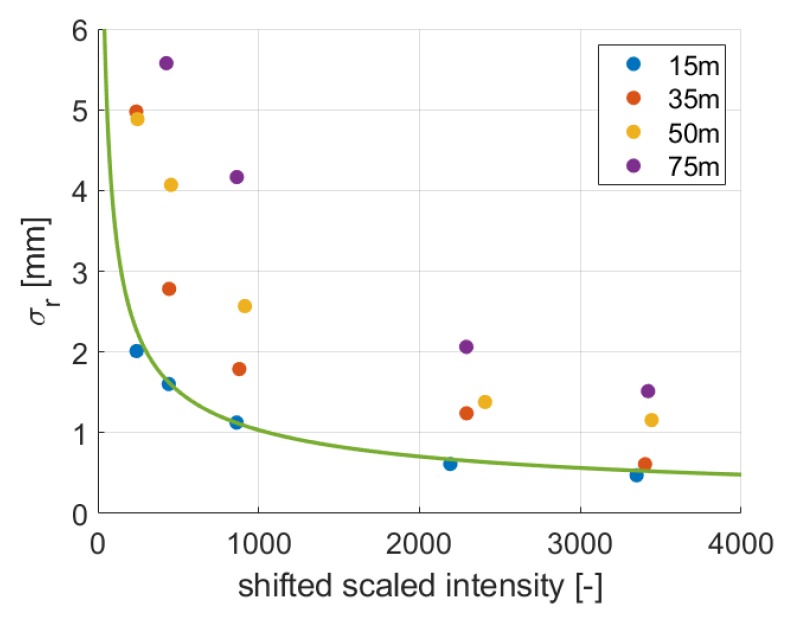
Range precision of samples with higher distances.

Figure 14 shows the estimated range precision of the different panels of the diffuse reflecting target. The different colors indicate the samples that are scanned with the same distance. The green line represents the estimated function from Figure 10, which includes samples scanned at distances of 8 m and 22 m. It is obvious that only the samples that were scanned from 15 m distance suit to the estimated curve. With higher distances, the standard deviation of the range increases. This shows that the intensity values of points that are measured with longer distances are amplified in order to keep the intensity of one object constant for different ranges. This also shows that it is not straightforward to use scaled intensities instead of the raw ones for estimating the intensity-based range precision.

This investigation limits the use of the estimated function to a maximum distance of around 20 m between scanner and target. The point clouds of the objects that are scanned with a longer distance do not follow the functional relation as it is modeled in Section 6.2. From these results, it is concluded that the effect, which comes along with the intensity amplification, is negligible for short ranges. For longer distances, new functions have to be estimated.

## 7. Discussion

In the two previous sections, it was investigated how the range precision of terrestrial laser scanners can be efficiently estimated even though no raw intensity values are provided by the manufacturer. Therefore, the methodology of [11] is extended. Finally, also samples that are not aligned perpendicular to the scanner can contribute to the estimation of the intensity-dependent range precision. Hence, simple cardboards can be used for the estimation. Thus, the setup is easy to build and can be measured quickly. With measurements from this setup, the range precision is modeled for the Leica HDS6100 and the Leica ScanStation P20 with scaled intensity values and raw ones if they are available. Table 1 shows a summary of all estimated parameters. The units of the parameters depend on the used intensity values. Inc denotes either the increments of the scaled Cyclone intensity values or the increments of the raw intensities from Z+F LaserControl. For the HDS6100, parameter *c* exists, whereas the adjustment for the P20 data does not have a significant third parameter. For this reason, *c* is not declared for the P20 in the table.

It is concluded that functions are now available for the Leica HDS6100 and the Leica ScanStation P20. However, if scaled intensities are utilized, the maximum range is restricted to 22 m. For this reason, the estimated function cannot be applied to the point cloud of the water dam since the ranges are too long (Section 1). Nevertheless, the range precision can be estimated for the other examples that were mentioned in the motivation.

**Figure 15:**
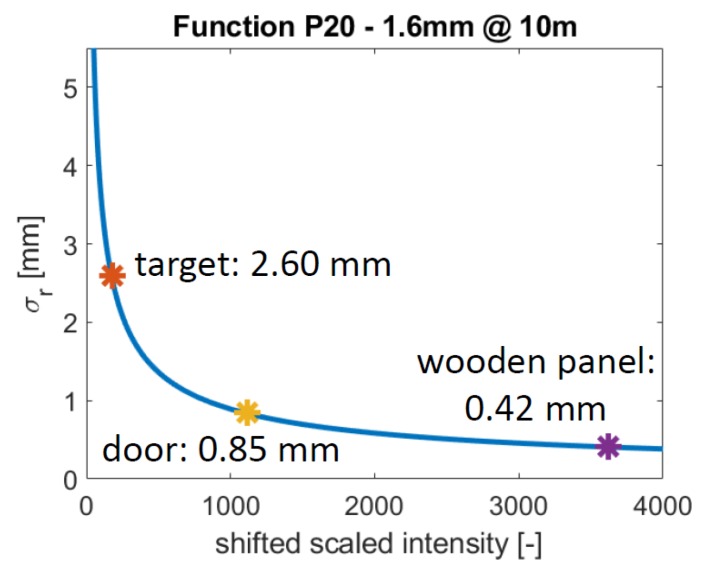
Corresponding range precision of the examples of Figure 1.

The objects were scanned with a resolution of 1.6 mm @ 10 m. Hence, the precision of the scan points is estimated with the parameters of this resolution. They are taken from Table 1. The intensity needs to be shifted by 2050 as explained in Section 6.2. Since the variation of the intensities within a point cloud is low, one point is randomly chosen in each point cloud. Figure 15 shows the range precision of these points. The range precision notably varies a lot. The most precise range measurements are obtained for the wooden panel, the least precise for the black target. This is not surprising considering their intensity values.

The black part of the target is the only surface of these examples that is known to be planar. For quick evaluation, a plane is estimated for this point cloud and the range precision is calculated as described in Section 3.1. A range precision of 2.58 mm is obtained, which only deviates 0.02 mm from the theoretic value (Figure 15). This means less than 1% of the actual range precision, which is acceptable and negligible.

## 8. Conclusions and Outlook

This study presents new approaches, which simplify the investigations for users of 3D TLS to analyze the range precision of their scanners. Since not all scanners can operate in 1D mode, and they do not supply raw intensity values, several aspects were examined, such as the estimation of the range precision with 3D points, finding the right measurement setup and the use of scaled intensity data. In order to compare raw and scaled intensity values, scans were collected with the Leica HDS6100 as both intensity types are available for this scanner. Furthermore, scans were taken with the Leica ScanStation P20 in order to model its intensity-dependent range precision. The following scientific contributions are gained from this study:

- This study introduced the estimation of the range precision by considering the range residuals of a plane adjustment and their standard deviation. As different observation groups are not weighted equally, the function can be properly estimated from samples with higher incidence angles. Thus, it is easier to get a wider range of intensity values and hence, this leads to a much quicker determination of the function. Furthermore, the proposed setup uses cheap cardboards, which are easy to install. Consequently, this simplifies the determination of the range precision of terrestrial laser scanners and makes it more efficient.
- It is demonstrated that the function, which models the relation between range precision and intensity, is applicable with raw intensity values, and likewise with scaled intensity values from Cyclone. However, this is only valid for shorter distances up to about 20 m. As the manufacturers modify the scaled intensities, this also influences the relation between range precision and intensity.

Based on these investigations, functions to model the intensity-dependent range precision could be determined for the Leica HDS6100 and the Leica ScanStation P20. However, the water dam is one example where the presented method reaches its limit as the measured distances are much higher than 20 m. The underlying reason for this is that the range precision cannot be determined independent from the distance if scaled intensities are used. On this account, this study needs further investigations in order to make the model applicable to each point cloud independent from the distance. Either the conversion between raw and scaled intensity values must be known or the manufacturer provides both types of intensities. Then, the scaled intensity values could be converted, and the function could be adjusted.

Another possibility is to build distance classes and to model a function for each distance class. This can be easily done by building the setup from Section 4.2 and placing the scanner at the desired distance. Hence, in the future, the range precision can also be determined for longer distances. However, attention needs to be paid to the size of the targets since the minimum number of points has to be retained. Furthermore, the results are only valid for the investigated scanners.

## Figures and Tables

**Table 1:** Estimated parameters of Equation (5) with standard deviation.

Scanner	a (STD)	b (STD)	c (STD)	$\rho_{\mathrm{ab}}$	$\rho_{\mathrm{ac}}$	$\rho_{\mathrm{bc}}$
(Intensity—Scan Rate/Resolution)	[mm/inc]	[–]	[mm]	[–]	[–]	[–]
**Leica HDS6100** **(raw—508 kHz)**	1970.32 (141.96)	−0.65 (0.02)	0.22 (0.02)	−0.99	0.92	−0.93
**Leica HDS6100** **(scaled—508 kHz)**	56.68 (3.27)	−0.69 (0.01)	0.27 (0.02)	−0.99	0.88	−0.92
**Leica ScanStation P20** **(scaled—0.8 mm @ 10 m)**	46.82 (2.21)	−0.55 (0.01)	–	−0.97		
**Leica ScanStation P20** **(scaled—1.6 mm @ 10 m)**	60.34 (3.67)	−0.61 (0.01)	–	−0.98		
**Leica ScanStation P20** **(scaled—3.1 mm @ 10 m)**	64.04 (5.06)	−0.64 (0.02)	–	−0.98		
